# Real-world ethics in palliative care: A systematic review of the ethical challenges reported by specialist palliative care practitioners in their clinical practice

**DOI:** 10.1177/0269216320974277

**Published:** 2020-12-10

**Authors:** Guy Schofield, Mariana Dittborn, Richard Huxtable, Emer Brangan, Lucy Ellen Selman

**Affiliations:** 1 Centre for Ethics in Medicine, Bristol Medical School, University of Bristol, Bristol, UK; 2 Paediatric Bioethics Service, Great Ormond Street Hospital, London, UK; 3 Health and Applied Sciences, University of West England, Bristol, UK; 4 Population Health Sciences, Bristol Medical School, University of Bristol, UK

**Keywords:** Ethical challenges, systematic review, palliative care, empirical bioethics, clinical ethics, bioethics

## Abstract

**Background:**

Ethical issues arise daily in the delivery of palliative care. Despite much (largely theoretical) literature, evidence from specialist palliative care practitioners about day-to-day ethical challenges has not previously been synthesised. This evidence is crucial to inform education and adequately support staff.

**Aim:**

To synthesise the evidence regarding the ethical challenges which specialist palliative care practitioners encounter during clinical practice.

**Design:**

Systematic review with narrative synthesis (PROSPERO registration CRD42018105365). Quality was dual-assessed using the Mixed-Methods Appraisal Tool. Tabulation, textural description, concept mapping and thematic synthesis were used to develop and present the narrative.

**Data sources:**

Seven databases (MEDLINE, Philosopher’s Index, EMBASE, PsycINFO, LILACS, Web of Science and CINAHL) were searched from inception to December 2019 without language limits. Eligible papers reported original research using inductive methods to describe practitioner-reported ethical challenges.

**Results:**

A total of 8074 records were screened. Thirteen studies from nine countries were included. Challenges were organised into six themes: application of ethical principles; delivering clinical care; working with families; engaging with institutional structures and values; navigating societal values and expectations; philosophy of palliative care. Challenges related to specific scenarios/contexts rather than the application of general ethical principles, and occurred at all levels (bedside, institution, society, policy).

**Conclusion:**

Palliative care practitioners encounter a broad range of contextual ethical challenges, many of which are not represented in palliative care ethics training resources, for example, navigating institutional policies, resource allocation and inter-professional conflict. Findings have implications for supporting ethical practice and training practitioners. The lack of low- and middle- income country data needs addressing.

**What is already known about the topic?**

- Evidence from other areas of healthcare practice demonstrates that the ethical challenges discussed in the literature do not always accurately reflect the range of challenges that healthcare practitioners experience in real-world practice.
- This phenomenon has not previously been systematically examined within palliative care.
- Improving our understanding of the ethical challenges faced by specialist palliative care practitioners is needed to support staff in their day-to-day practice and to underpin evidence-based ethics training programmes in palliative care.

**What this paper adds**

- This systematic review identified ethical challenges in six themes: application of ethical principles; delivering clinical care; working with families; engaging with institutional structures and values; navigating societal values and expectations; and the philosophy of palliative care.
- The range of ethical challenges faced by specialist palliative care practitioners exceeds the breadth of those detailed in palliative care textbooks and ethics resources.
- The review found no data from low- or low middle-income country settings.

**Implications for practice, theory or policy**

- The broad range of ethical challenges identified suggests that training programmes and core texts in the field should expand their coverage to better support practitioners.
- As most practitioners described highly context-based ethical challenges, tools that specifically include contextual data may be more appropriate when practitioners analyse their own cases.
- The lack of data from low- and middle- income countries needs addressing as these are the settings with the highest levels of palliative care need, and many identified ethical challenges are context-specific and therefore may not be transferable between settings.

## Introduction

In published literature, palliative care is associated with ethical challenges across varied aspects of clinical care.^1–4^ Challenge areas include, for example, withdrawing and withholding of interventions,^5^ dignity and quality of care,^6^ respect for autonomy^7^ and palliative sedation.^8,9^ However, there is evidence from other areas of healthcare practice that the ethical challenges examined within theoretical literature do not accurately reflect the range of the dilemmas that healthcare workers report experiencing in real-world practice.^10–12^ Whilst this mismatch between lived experience and the theoretical academic literature has not previously been systematically examined within palliative care, there is some evidence suggesting it applies.^13–15^ Hermsen and ten Have,^13^ for example, compared the ethical challenges reported by specialist palliative care providers with those found in the palliative care literature. They found 14 reported ethical challenges had no accompanying literature, and two topics with significant literature, including engaging with ethics committees, which were not reported in practice.^13^

To the authors’ knowledge there is no project that has systematically collated the range of ethical challenges that are encountered within palliative care. Addressing this knowledge gap is important for the field going forward as training in the ethical aspects of palliative care is recognised as a priority and often requested by pracitioners.^4,16^ A thorough understanding of the ethical context practitioners work within is needed if educators are to generate evidence-based curricula that reflect real world contexts. Education activities can benefit from a robust grounding in the real-world experiences of learners as the relevance of educational material is a key factor in adult learner motivation,^17^ and processing new material in relation to prior experiences contributes to learning efficiency.^18^ Also importantly, as palliative care provision expands across the globe, there is a need to understand the nature and pattern of ethical challenges in differing global contexts. The palliative care global health literature currently contains little empirical engagement with ethical challenges within the field.^19–21^

We aimed to review and synthesise the literature to answer the research question: what ethical challenges do those working in specialist palliative care report experiencing in clinical practice?

## Methods and analysis

We conducted a systematic review to identify and summarise empirical data on the ethical challenges specialist palliative care practitioners report experiencing. We used narrative synthesis, following the iterative framework from Popay et al.,^22^ adapted for a review which does not focus on an intervention. The integration of themes and content was guided by Thomas and Harden’s^23^ ‘thematic synthesis’ approach.

The review protocol was designed and reported with reference to Preferred Reporting Items for Systematic Reviews and Meta-Analyses Protocols (PRISMA-P).^24^ We follow the PRISMA reporting guidelines for systematic reviews.^25^ The protocol was prospectively registered with PROSPERO (CRD42018105365)^26^ and published open access.^27^ There were no deviations from the published protocol, outlined below.

## Eligibility criteria

Inclusion and exclusion criteria are summarised in Table 1. As the commonly-used participants, interventions, comparisons, outcomes and study design (PICOS) system is not suitable for argument-based or empirical ethics reviews,^28^ we used Strech et al.’s proposed adaptation: the Methodology, Issue, Participants (MIP) system.^28^

**Table 1.** Inclusion and exclusion criteria.

	Inclusion criteria	Exclusion criteria
Types of participants	Study participants are specialist palliative care practitioners (SPCPs) working in a patient care role. We define SPCPs as people working in, or for, a healthcare setting whose main focus is on delivering palliative care (as opposed to clinical contexts where palliative care forms part, but not the main focus, of the care provided).	Participants who undertake palliative care tasks as part of their role (e.g. oncologists), but who do not specialise in providing palliative care and do not have palliative care as the main focus of their role.
	This may include (but is not limited to) nurses, doctors, occupational therapist, physiotherapists, dieticians, speech and language therapists, psychologists, other allied health professionals and chaplains.	
	Studies with a mixed population where SPCP participants’ data are separately presented and can be extracted will be included.	
Context	All geographical settings and all clinical settings where specialist palliative care (SPC) is delivered will be included.	Studies conducted in settings in which SPC is not being delivered.
Issues	The range of ethical challenges that are reported as experienced by SPCPs during clinical delivery of palliative care.	Studies that utilise survey tools with pre-selected ethical dilemmas that have not been inductively derived based on evidence from SPCPs, and studies that investigate a single aspect of palliative care only will be excluded.
	The definition of ‘ethical challenges’ will be intentionally kept broad to capture the maximum number of examples. It includes but is not limited to terms such as ethical issues, moral challenges, moral dilemmas, values, good/bad, right/wrong. Ethical challenges can be labelled as such either by authors or participants.	These study designs are excluded as they proceed from an a priori assumption that their selected issues are relevant. They therefore do not contribute to an inductive exploration of the breadth and type of ethical challenges facing practitioners.
Methodologies	Empirical studies examining, using inductive methods, the ethical challenges reported by SPCPs in their clinical practice. These may include qualitative studies, mixed methods studies (e.g. surveys with free-text responses) or quantitative studies using questionnaires derived inductively through consultation with SPCPs.	Studies not reporting inductively derived empirical data. These may include studies using questionnaires which include ethical challenges selected a priori, or single-issue studies focussed on an ethical challenge selected a priori by the researchers.
Timeframe	Any time frame up until the search date will be included, contingent on the inception dates of the databases included in the search.	
Type of publications	Peer-reviewed journal publications of empirical research. Papers in any language will be included, with findings translated into English where necessary.	Where no full text is available through the university subscription, study authors will be contacted for full text. If there is no response within 2 weeks the study will be excluded. The following will also be excluded: - Conference abstracts; however, authors will be contacted for further data/publications. - Editorials, letters, or comment/opinion pieces. - Review articles. Reviews will be used for identification of primary research only. - Book sections.

The review included peer-reviewed inductive studies which identify ethical challenges practitioners face in their real-world clinical practice, or secondary analyses of such data. Following Creswell and Plano Clark, inductive data, for the purpose of this review, is defined as that which derives from data collection efforts that occur independently from any attempt to validate a particular theory or hypothesis.^29^ Studies that explored single topics in specialist palliative care practice selected a priori by researchers were excluded, as were studies that used a pre-determined list of ethical challenges. Whilst data from both these study types might contribute to describing ethical challenges in palliative care, both study types pre-suppose the presence of the challenges they focus on within the experiences of palliative care practitioners, which, as demonstrated by Hermsen and ten Have, may not be the case.^13,27^

To minimise bias and the omission of relevant international data, there were no language, geographical or timeframe restrictions, although the databases searched were in English.

## Search strategy

We identified databases to search in conjunction with subject information specialists and by identifying which databases indexed journals containing key papers known to the research team. The following databases were searched on 19th December 2019: MEDLINE (Ovid interface, 1946 onwards), Philosopher’s Index (OVID interface, 1940 onwards), EMBASE (OVID interface, 1980 onwards), PsycINFO (OVID interface, 1806 onwards), LILACS (http://lilacs.bvsalud.org/en/, 1982 onwards), Web of Science (Clarivate interface, 1900 onwards) and CINAHL (EBSCO interface, 1937 onwards). Medline search terms (Table 2) were adapted for the other databases.

**Table 2.** Medline search strategy.

1 Ethics/ 2 Ethics, Nursing/ 3 Ethics, Medical/ 4 Ethics, Clinical/ 5 exp Ethics, Professional/ 6 Bioethics/ 7 moral*.tw. 8 ethic*.tw. 9 1 or 2 or 3 or 4 or 5 or 6 or 7 or 8 10 Palliative Care/ 11 Palliative Medicine/ 12 Terminal Care/ 13 Hospice Care/ 14 Hospices/ 15 ((end of life or terminal*) adj3 (ill* or care)).tw. 16 palliat*.tw. 17 hospice*.tw. 18 10 or 11 or 12 or 13 or 14 or 15 or 16 or 17 19 9 and 18 20 exp animals/ not humans/ 21 exp Animals, Laboratory/ 22 exp Animal Experimentation/ 23 exp Models, Animal/ 24 (rat or rats or mouse or mice or rodent*).ti. 25 20 or 21 or 22 or 23 or 24 26 19 not 25 27 exp ‘Surveys and Questionnaires’/ 28 survey*.mp. 29 question*.mp. 30 or/27-29 31 (((‘semi-structured’ or semistructured or unstructured or informal or ‘in-depth’ or indepth or ‘face-to-face’ or structured or guide) adj3 (interview* or discussion* or questionnaire*)) or (focus group* or qualitative or ethnograph* or fieldwork or ‘field work’ or ‘key informant’)).ti,ab. or interviews as topic/ or focus groups/ or narration/ or qualitative research/ 32 30 or 31 33 26 and 32

Initial scoping searches suggested that the search terms would return over 25,000 highly varied records, and that relevant inductive studies would use qualitative methods or mixed-methods surveys incorporating free-text responses. To capture the most relevant records we therefore used peer-reviewed methodological filters (Supplemental File 1) to refine the search results. The methodological filters were initially identified via the InterTASC Information Specialists’ Sub-Group Search Filter Resource.^30^ Sentinel research outputs, known to the researchers prior the review, were tracked through the filter process and all were retrieved.

Reference lists of included papers were hand-searched. Corresponding authors of papers meeting the inclusion criteria were contacted and asked if they recommended other published work for review. Authors of conference abstracts were contacted for peer-reviewed data or follow-up publications if available, but no additional papers were identified. A grey literature search was not conducted. Cook et al. demonstrated that extensive grey literature searching did not benefit the review content of a palliative care systematic review despite requiring significant resources.^31^

Search results were exported to, and collated and de-duplicated in, Endnote X9.2.^32^

## Selection process

Retrieved records were screened at title and/or abstract level by GS. A second researcher (MD) independently screened a random sample of 10%.^33^ Differences in screening between GS and MD were discussed within the research team to clarify and refine inclusion/exclusion criteria. Study authors were contacted when further information was required. Contested papers were examined by a third reviewer (LS). The full texts of potentially eligible records were retrieved and independently assessed for eligibility by GS and MD.

## Data extraction and management

Data extraction was undertaken independently by GS and MD, using a pre-piloted data extraction form. No data disagreements emerged. Data items extracted from included studies were: (1) citation details including title, publication year and journal; (2) study setting, methods, participant characteristics, sample size; (3) specified definition/conceptualisation of ethical challenges; (4) the study’s key findings, themes and sub-themes; and (5) sources of potential bias including funders and evidence of reflexivity. There was no missing data.

## Data synthesis

We conducted an adapted narrative synthesis following the relevant framework stages described by Popay et al.,^22^ namely: developing a primary synthesis, exploring relationships within and between studies, and assessing robustness of the synthesis. Preliminary synthesis development included identifying and tabulating both textual descriptions of studies and study participant-derived data. Thematic synthesis based on Thomas and Harden’s framework^23^ was then used, iteratively utilising the three stages of this approach: line-by-line coding of the text presented in the Results sections of the papers; development of descriptive themes; grouping and organisation of descriptive themes into higher-level analytical themes. Relationships within and between studies were explored (informed by concept mapping), including a focus on possible patterns related to study or participant characteristics such as geographical location, care setting and professional background. GS led the synthesis with regular discussions with MD and further discussions with EB, RH and LES until consensus around identified themes was reached. The robustness of the synthesis was enhanced through adopting this highly collaborative approach and the use of systematic methods to assess study quality (MMAT 2018)^34^ and the final synthesis output (GRADE-CERQual).^35^ As the review aimed to map the ethical challenges reported by specialist palliative care practitioners, we did not carry out theory development.

## Risk of bias (quality) assessments

Scoping searches suggested that relevant studies would use qualitative and mixed-methods designs. To allow comparison of study quality, the Mixed-Methods Assessment Tool (MMAT) 2018^34^ was used, with each study being scored independently by two reviewers (GS and MD). The MMAT focuses primarily on the methodological aspects of assessed studies, which aligns with the GRADE-CERQual recommendations for choosing a quality assessment tool.^36^

To assess the quality of the review findings we applied GRADE-CERQual,^35^ which provides a systematic framework for assessing confidence in individual review findings, based on consideration of four components: (1) methodological limitations, (2) coherence, (3) adequacy of data and (4) relevance.

## Results

**Figure 1.**
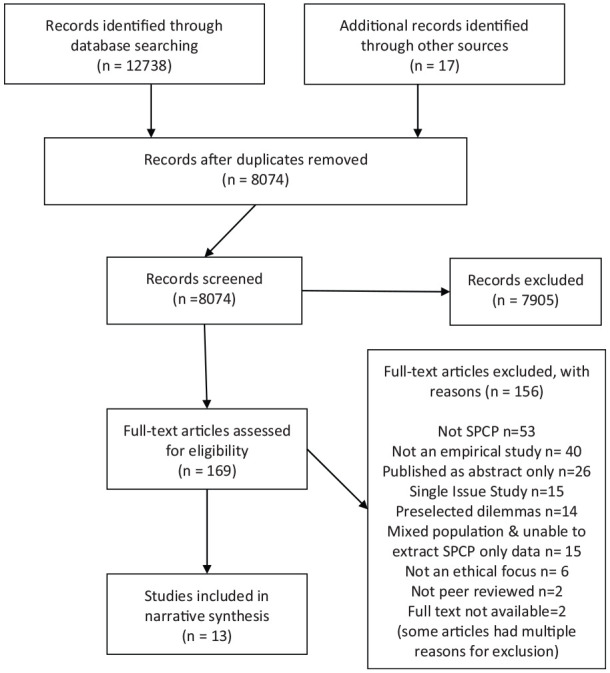
PRISMA flowchart.

After de-duplication, the electronic searches, hand-searching and author contact identified 8074 individual records. A total of 7905 records were excluded at title and/or abstract level. About 170 abstracts were available only in Spanish or Portuguese and were assessed by MD, a native Spanish speaker also proficient in Portuguese. Only one record, published in Mandarin from Taiwan, was retrieved that did not have an English, Spanish or Portuguese language abstract. This was assessed by a native Mandarin speaker who was briefed on the inclusion criteria for the review and was excluded at the abstract stage. About 169 records were screened at full text, with 13 meeting the inclusion criteria (Figure 1). Reference lists of these papers were hand-searched. Ten additional records were found but excluded at the abstract stage.

All papers were published in English. The reported studies represented research from multiple international settings: Brazil,^37,38^ Canada,^39^ Germany,^40,41^ Mexico,^15^ the Netherlands,^13^ Portugal,^42^ Sweden,^43^ Taiwan^44^ and USA.^45–47^ Studies were published between 2000 and 2017; see Table 3 for details.

**Table 3.** Included study details.

Authors	Year	Study title	Country	Study design	Study population	Study setting – category	Adult or paediatric	Pre-definition of ‘ethical challenge’?	High level themes
de Andrade et al.^37^	2006	Palliative care and bioethics: study with assistance nurses	Brazil	Mixed Methods – Cross Sectional Survey	28 nurses	IP hospital	Adult	No	2
Bezerra do Amaral et al.^38^	2012	Ethic and bioethic dilemmas on palliative care for hospitalised elderly: nurses’ experience.	Brazil	Qualitative – Semi-Structured Interview	10 nurses	IP hospital	Adult	No	4
Cheon et al.^45^	2015	Ethical issues experienced by hospice and palliative nurses	USA	Mixed Methods – Cross Sectional Survey	129 nurses	Unclear	Mixed	No	6
Chiu et al.^44^	2000	Ethical dilemmas in palliative care: a study in Taiwan	Taiwan	Mixed Methods – observation and survey	Healthcare workers caring for 246 patient care admissions	IP hospital	Adult	No	9
Dennis et al.^46^	2014	Ethical dilemmas faced by hospice social workers.	USA	Qualitative – Semi structured interview	14 social workers	Mixed	Mixed	No	4
Guevara-López et al.^15^	2015	New frontiers in the future of palliative care: real-world bioethical dilemmas and axiology of clinical practice.	Mexico	Mixed Methods – Cross sectional survey, semi-structured interview	30 physicians	Mixed	Adult	Yes	17
Hermsen and ten Have^13^	2003	Moral problems in palliative care practice: a qualitative study	The Netherlands	Mixed Methods – observation and bibliographic analysis	Hospice	IP hospice	Adult	No	5
Hernández-Marrero et al.^42^	2016	Ethical decisions in palliative care: Interprofessional relations as a burnout protective factor? Results from a mixed-methods multicentre study in Portugal	Portugal	Mixed Methods – cross sectional survey and semi-structured interviews	18 physicians and 70 nurses	Mixed	Adult	No	n/a
Hold^47^	2017	A good death: Narratives of experiential nursing ethics	USA	Qualitative – Interview	6 nurses	OP hospice	Adult	Yes	3
Salloch and Breitsameter^40^	2010	Morality and moral conflicts in hospice care: results of a qualitative interview study	Germany	Qualitative – Semi structured interviews	2 nurses, (4 volunteers (2 active, 2 retired))*	IP hospice	Adult	No	2
Sandman et al.^43^	2017	Developing organisational ethics in palliative care: A three-level approach	Sweden	Qualitative – participatory action research, focus groups	15 nurses, 10 assistant nurses	IP hospital	Adult	No	6
Towers et al.^39^	2013	Ethical issues in palliative care. Views of patients, families, and non-physician staff	Canada	Qualitative – Semi structured interview	(Patients, relatives)*, 14 Nurses, three Psychologists, three Staff physicians, one Occupational therapist, one Music therapist, one Pastor, one Resident physician, one Unit clerk	IP hospice	Adult	No	3
Walker and Breitsameter^41^	2015	Ethical decision-making in hospice care	Germany	Qualitative – Observation and semi-structured interviews	14 nurses, three geriatric nurses, one social worker	IP hospice	Adult	No	3

* In these studies it was possible to separate data from different participants.

IP: in-patient; OP: out-patient.

### Study quality assessment

**Figure 2.**
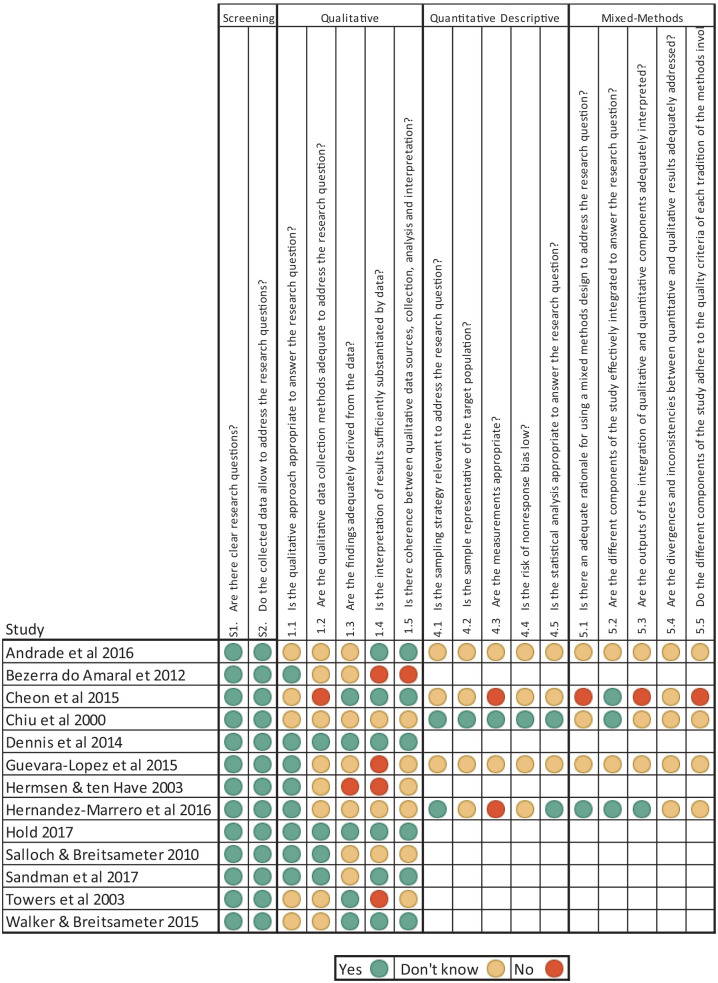
MMAT evaluations for included studies.

All studies were dual evaluated by GS and MD using the MMAT 2018.^34^ One study, de Andrade et al.,^37^ required discussion with a third reviewer (LS) as to which sections of the tool were appropriate. There were no disagreements in assessment. See Figure 2 for the MMAT evaluations.

### Findings quality assessment

Summary GRADE-CERQual review findings are presented in Table 4. For all findings, all contributing studies were assessed as directly relevant using GRADE-CERQual guidance on assessing relevance.^44^ Therefore, we used the same approach used in other GRADE-CERQual assessment areas^35,48–50^ to assess bias towards particular geographical location, professional background or timeframe. The GRADE-CERQual assessments suggest that reasonable confidence can be taken that the ethical challenges we identified exist in day-to-day practice. For the full assessment table, see Supplemental File 2.

**Table 4.** GRADE CERQual findings summary table.

Review findings by theme	Studies contributing to the review finding	CERQual assessment of confidence in the evidence	Rationale for CERQual assessment
1. *Application of Ethical Principles.* Ethical challenges that relate to practitioners applying bioethical principle to clinical situations			
1.1 *Autonomy*. Challenges related to the bioethical principle of autonomy	^13,37–41,45,46^	High confidence.	This review finding was present in 8/13 included studies. Despite methodological concerns the data in this finding was consistent across the included studies. The authors have high confidence challenges related to autonomy feature in every day palliative care practice.
1.2 *Dignity*. Challenges that engage any variant of the concept of dignity	^37,39,40,46^	High confidence.	Despite a rating of moderate concerns for coherence, and adequacy, this is a broad descriptive review and the authors have confidence that SPCPs engage with dignity within their everyday ethical challenges. The nature of dignity is highly contested in bioethics and the review findings reflect this.
1.3*Truth Telling*. Participants reported ethical challenges related to the ethical principle of truth-telling. This focuses around whether it was appropriate to discuss both the terminal nature of a diagnosis and/or prognosis to both the patient and to family members. It also covered whether it may be appropriate to withhold information from patients, either because of clinician beliefs regarding harms, or the wish of the families.	^13,15,38,39,42,43–47^	High confidence.	This finding is contributed to by most of the studies included in the review. There is a high level of coherence between the individual study findings. The authors are confident that ethical challenges related to truth telling form part of the network of challenges faced by practitioner in day to day clinical practice.
1.4 *Doctrine of Double Effect.* Challenges that relate to scenarios where the doctrine of double of effect is thought to have been enacted, usually around the prescription and effects of opioids.	^13,15,38,41^	Moderate confidence.	The authors are moderately confident that SPCPs felt that they experienced ethical challenges engaging with the doctrine of double effect as described in the contributing studies. There is a lack of detailed description in the primary data and the locations of the source studies, in both geography and in time, may suggest a variance in how widely this is experienced.
1.5 *Equity in care*. Ethical challenges that engage with promoting equity in the delivery of palliative care across multiple patients.	^13,37,43^	Moderate confidence.	The authors assessed that whilst there are multiple areas of concern for this finding, it must be viewed in the context of this review being very descriptive in nature. Detailed understanding of the concept is not required for the review to meet its aims. Therefore, the authors have moderate confidence that issues regarding equity in care are experienced in clinical practice amongst SPCPs.
1.6 *Fidelity*. Ethical challenges exist that relate to the professional ethics value of fidelity, which is a value described as one of seven that make up the seven ethical principles of nursing and public health ethics.	^47^	Moderate confidence.	The authors felt that although this finding was derived from a high quality recently published study the paucity of primary data and the narrow range of participants (nurse only) allowed, at best, for only moderate confidence in this finding.
2. *Delivering Clinical Care.* Ethical challenges embedded in clinical decision-making and bedside care			
2.1 *Clinical care and decision-making*. Ethical challenges that relate to the provision of specific clinical interventions rather than more general goal-based decision-making. There is some unavoidable overlap with goals of care (2.4).	^13,15,38–46^	High confidence.	The authors assess this finding as high confidence. The variety of challenges grouped that made up the sub findings were describes across multiple contributing studies, represented all geographical and income settings of the included studies and represented views from a broad range of participant professional backgrounds.
2.2 *Confidentiality.* Ethical challenges relating to clinical confidentiality.	^37,39,43^	High confidence.	Despite the range of moderate concerns, the authors have assessed a high confidence in this finding. The concept of confidentiality is usually well understood amongst healthcare professionals and in the literature, and therefore can be understood with thinner data than might be required for other findings.
2.3 *Goals of care.* This related to decision-making regarding broader aspects of clinical care, as compared to the more focused medical sub-theme that focuses on individual clinical interventions. Broader topics included dilemmas concerning overall therapeutic aims and strategies, particularly around clinical decision-making moving from disease modifying to symptom management only, withdrawing and withholding, treatment proportionality and assessment of futility, and patient’s preferred place of care and death.	^13,15,37–47^	High confidence.	The authors have high confidence in this finding. The finding is contributed to by all included papers with a high level of coherence in the high-level nature of the challenges. Deeper concepts within the challenges do slightly differ in the deviant case study but that goals of care is a challenge is not disputed.
2.4 *Mental capacity.* Participants reported ethical challenges were experienced relating to both the assessment of mental capacity and the role and choice of proxy decision makers should capacity be assessed as being lost in a specific instance.	^13,41,42,45,46^	High confidence.	The authors assess this finding as high confidence. The contributing studies represented the participants across all included studies as a whole in both range of professional backgrounds and geographical context. The contributing studies had good levels of coherence between themselves allowing coherence with the review finding.
2.5 *Communication with patients and their families.* Reported challenges included; perceived quality of information given, including managing conflicting information from multiple teams, poor availability of staff to facilitate communication, communication between professionals, and differences in the cultural frames of reference within conversations.	^13,15,39,42,43,45^	Moderate confidence.	The authors rate this finding as only moderate confidence as, although it is clear there is an area of challenges described that is distinct from other findings areas (such as truth telling above), there is a thinness in the data regarding the content of this finding. It may be that richer data described a different range of content for this finding as some contributing primary study data may have been re-coded to other findings, altering the content of this one.
3. *Working with Families.* Multiple ethical challenges are experienced that are derived from working alongside families as they support their relatives, from care of the family members themselves, or as a whole.			
3.1 *Care and support for the family.* Challenges related to the care/support of patients’ families and children. This finding focused on supporting patients’ children, particularly where the professionals view on the best way of supporting the child differed from the patient or family member, and separately support for family on the ward in terms of bedding/food etc.	^39,43,46^	Moderate confidence.	The authors rate this finding as moderate confidence. Derivation from high quality studies supports its inclusion at the finding level, but the thinness of the data and the disparity between the studies’ results that whilst there is confidence this is a challenge area, the exact nature of these challenges is not reliably discernible from the primary data.
3.2 *Family as decision makers.* Challenges arising from when families requested clinical interventions that the health professionals thought were not in the patient’s best interests or insisted on the withholding of diagnostic or prognostic information from the patient. These were particularly pronounced in situations where the patient lacked capacity to express their own wishes.	^39–41,43,45–47^	High confidence.	The authors have high confidence in this finding. The coherence between the contributing studies, and their high methodological assessment, is supportive of the finding being one of the ethical challenge areas that are experienced by palliative care practitioners.
3.3 *Genetics.* Practitioners report challenges when supporting or advising patients and their families about genetic elements of conditions that are potentially hereditable.	^13^	Moderate confidence.	This finding is rated as moderate confidence. The authors have moderate confidence that there is a collection of challenges related to genetics in the daily practice of specialist palliative care. However, the exact nature of this challenge cannot be determined from the data contained within the contributing study.
3.4 *Privacy*. Challenges around families being present for care episodes that the practitioners felt was not appropriate.	^39,43^	Moderate confidence.	This finding is assessed as moderate confidence. The studies that contribute to it scored well on methodology, and the finding is coherent. There is however limited detailed primary data and so again as this is a very descriptive review the level of confidence is higher than it might have been should an explanatory power be required.
4. *Engaging with Institutional Structures and Values.* Ethical challenges that engage with values held by other professionals, professional groups or healthcare institutions			
4.1 *Conflict with institutional policy.* In this finding ethical challenges were experiences when engaging with institutional and organisational policies that impacted on the participants care of the patients.	^13,15,43,46,47^	High confidence.	This finding is assessed as high confidence. The contributing studies are clear in identifying that engaging with institutional policies create ethical challenges in the care of individual patients. The content of the challenges varies between studies, but the overarching concept is rich enough for a descriptive review and for an assessment of high confidence.
4.2 *Institutional resource allocation.* This finding focused around internal resource allocation. This is variously manifested; risks from delays in admissions due to lack of resources and staff availability, pressure in institutions for limited lengths of stay and deficiencies in the quality of care resulting from perceived understaffing.	^39,44^	High confidence.	The authors judge this finding has having high confidence. The coherence between the contributing studies and the range of the contributing studies supports the finding that resource allocation creates ethical challenges for individual healthcare practitioners.
4.3 *Conflict between healthcare staff*. Ethical challenges that conflict between individual palliative care team members, conflict between members of the multidisciplinary team that the participants felt was related to differing professions prioritising different ethical principles, and conflict with other clinical specialties or disciplines.	^15,39–41,43,45–47^	Moderate confidence.	The authors assess this finding as moderate confidence. The low level of coherence between the contributing studies in both the detail and more general content increases the risk that although challenges of this nature exist, this review may not accurately capture their content.
5. *Navigating Societal Values and Expectations.* Ethical challenges that intersect with values in society more broadly, situated beyond the healthcare practitioner or healthcare institution.			
5.1 *Assisted Dying*. Ethical challenges related to how to handle patient’s requests for assisted dying, rather than the more general question about whether it should be available as an option. This finding also explored participant concerns of the border between appropriate opioid prescribing and hastening death.	^13,15,39–41,46^	High confidence.	The authors assess this finding as high confidence. The range of contributing studies, and level of agreement between them in the formulation of the challenge support this assessment.
5.2 *Conflict with wider societal rules, regulations or laws.* This finding pertains to a conflict of duties for the healthcare professional when a patient’s autonomous choice conflicts with what is permitted under the licencing system of healthcare providers, or the law more generally.	^46^	Moderate confidence	The authors assess this as moderate confidence. The finding derives from a single high-quality study. This rigour in method helps support the inclusion of the finding in this descriptive review. What is less certain is the exact nature of the challenges, and how this may change across professional groups and service locations.
5.3 *Access to specialist palliative care*. Inequitable access to specialist palliative care services leading to perceived harms to the affected individuals. This finding is tied to the bioethical concept of justice.	^42^	Low/moderate confidence.	The authors assess low/moderate confidence in this finding. Whilst the single contributing study is clear in locating the source of the challenge, it lacks enough detail to understand it in more detail that title only.
5.4 *Cultural and spiritual considerations.* Ethical challenges were identified that were based in a possible lack of mutual frames of reference, language, and cultural and spiritual practices regarding death and dying.	^15,39,43^	Low confidence.	The authors assess this finding as low confidence based on the data from the contributing primary studies. That ethical challenges may be based in cultural and spiritual aspects of care is clearer, but the specific description of this finding as derived from the thin underlying data is potentially unreliable.
5.5 *Suicide.* Ethical challenges that relate to how practitioners should respond to patient reports of suicidal ideation and planning.	^15,46^	Low confidence.	The authors assess this finding as serious concerns. The thinness of the data that contribute to this finding, and the inclusion of only 2 studies results in low confidence in the content of the finding, as it is potentially likely that more detailed exploration of the topic would generate conflicting and contrasting data points.
6. *The Philosophy of Palliative Care.* Ethical challenges relating to how provision of particular approaches to care might be judged from differing viewpoints related to the nature and aims of palliative care.			
6.1 *Philosophy of palliative care*. Participants reported challenges related to the very nature of the aims of the field of specialist palliative care itself.	^13,38,40,41,43^	Moderate confidence.	The authors have moderate confidence in this finding. The deeper details are less clear but that there are challenges that are the result of more abstract debates about the nature and role of palliative care filtering down to the bedside seems clear.

### Themes

The ethical challenges reported in the included studies were organised into six major themes with sub-themes (Table 4): the application of ethical principles; delivering clinical care; working with families; engaging with institutional structures and values; navigating societal values and expectations; and the philosophy of palliative care.

Overall, ethics was felt to be a major feature of participants’ roles, adding difficulty and complexity.

*‘Again and again, it’s morals and ethics that make things so difficult. It’s not the job. It’s those two packages that can sometimes be quite a burden’* (Nurse, Germany, p590)^40^

Challenges were widely distributed across the included studies with relation to geography, professional background and publication date. There were very few differences in the patterns of challenges across differing country settings. An exception is that ethical challenges focusing on use of alternative medicine were reported only in a study from Taiwan, where there are high rates of patient use of traditional Chinese medicine.^44^ Comparison of challenges between different professional backgrounds demonstrates only a few unique challenges. In one study physicians identified ethical challenges in clinical decision-making regarding antibiotics prescribing and fluid replacement.^15^ Engaging with the principle of fidelity was identified only in one nursing study.^47^ Conflict with wider societal regulations and professional licensing was documented only in a Canadian study that focused on social worker experiences.^46^

#### Application of ethical principles

Findings in this theme relate to practitioners’ reflections on established bioethics principles within their clinical work. Sub-themes were autonomy; dignity; truth-telling; doctrine of double effect; equity in care and fidelity.

##### Autonomy

This finding was reported by 8/13 studies.^13,37–41,45,46^ Participants reported multiple related challenges: how best to support patients in making autonomous decisions and protect patients from coercive influences including from family members, how to respond when patients made decisions that the practitioner judged would increase harm/risk to the patient^41,46^ or to others^41^ or that conflicted with professional judgement of what was in their best interests and therefore beneficence,^40,41,46^ or with the personal values of the staff caring for them.^40,46,47^

*‘I am responsible for the person who entrusts himself to me, and I need to understand his wishes. That doesn’t mean that I think everything is right, but that’s my problem.’* (Nurse, Germany, p323)^41^

##### Dignity

Dignity was a focus of challenges in three studies.^37,40,46^ For some participants dignity was tied to patient autonomy and the challenges of respecting it,^37,40,46^ with one participant describing it in terms of patients’ rights to choose riskier options, ‘*the dignity of risk*’ (Social Worker, USA, p957)^46^ Participants in two studies felt that dignity was also related to empathetic and equitable terminal care and not leaving patients alone at the end of life.^37,40^ In a German study, participants described how euthanasia was directly opposed to a death with dignity, and felt that patient dignity was supported by organisational and practitioner opposition to the provision of euthanasia.^40^

##### Truth-telling

Challenges related to truth-telling were reported in 10/13 studies.^13,15,38,39,42–47^ Challenges related to a patient’s diagnosis,^15,38^ or prognosis, particularly if this was judged to be short,^38,39,44,46^ and occurred when either practitioners or families decided whether it was appropriate to inform a patient or to withhold this information. Participants in several studies identified the conflict with patient autonomy in the case of withheld information.^13,38,42,45,46^ Other dilemmas involved probity or veracity, describing administration of covert medication,^46^ or the inclusion of misleading information on medication requisitions to alter whether the patient, hospice or insurance company paid for them.^47^

*‘We have had several families who don’t want the patient to know the diagnosis, the prognosis, or that they are in hospice’.* (Nurse, USA, p10)^45^

##### Doctrine of double effect

4/13 included studies^13,15,38,41^ reported ethical challenges related to the administration of medication to relieve symptoms and participants’ concern that this may shorten life.

*‘There was one patient who was in a lot of pain and had morphine prescribed . . . [and] administered and the patient went (. . .), the blood pressure was inaudible, and slowly the patient deceased’* (Nurse, Brazil, p20).^38^

##### Equity in care

This finding derives from three studies.^13,37,43^ Participants encountered challenges when trying to treat patients equitably, with ‘fair treatment’ and without discrimination and with respect to their rights.^37^

*‘Do not discriminate against the patient at any time, always seek to provide an equal service’* (Nurse, Brazil, p4926)^37^

Two studies identified found that clinicians’ judgements of a patient’s behaviour as ‘good’ or ‘bad’ could affect equity in care and judgements of this nature were felt to be ‘problematic,’ impacting practitioners’ relationships with patients.^13,43^ In a Dutch ethnographic study the observer describes a patient that regularly ‘flies into a rage’ and uses abusive language and how because of this the nurses have trouble feeling ‘sympathy’ for the patient and her situation (p268).^13^

##### Fidelity

Fidelity refers to the value of remaining true to a profession’s values and focus on the patient. Nurses in the USA-based contributing study reported that this principle is challenged when other stakeholders interfere with the nurses’ commitment to patients.^47^

#### Delivering clinical care

Findings in this theme focused on dilemmas surrounding the provision of patient care, including clinical decision-making. Five sub-themes were identified: clinical care and decision-making; communicating with patients and families; confidentiality; goals of care; and mental capacity.

##### Clinical care and decision-making

This sub-theme relates to specific clinical interventions or decisions (Table 5), some of which overlap with challenges reported in *Goals of care*.

**Table 5.** Clinical care and decision-making sub-themes.

Ethical challenge	Description
Administration of antibiotics^15^	Appropriate use of antibiotics, particularly in end of life care.
Advance directives^13,40,45^	Challenges implementing advance directives, particular when family requests may contrast with the directive.
Bloods transfusions^15,44^	Appropriateness of blood transfusions.
Deactivation of permanent pacemakers^45^	Appropriateness and timing of deactivation of cardiac pacemakers.
Do-not-resuscitate decision-making^13,43^	Decision-making about appropriateness of cardiopulmonary resuscitation.
Electrolyte management^15^	Clinical decision-making about management of abnormal electrolyte results.
Hydration and nutrition^13,15,38,40,41,44–46^	A broad range of challenges related to the provision, withdrawal and withholding of routine as well as clinically-assisted oral nutrition and hydration. Also includes issues of force feeding.^13^
Investigations^43^	Decision-making regarding which clinical tests are appropriate.
Sedation incl. palliative/terminal sedation^13,15,39–42,44,45^	Ethical dilemmas concerning use of sedatives for either symptom control or continuous sedation until death.
Symptom management^13,39,45^	Appropriate use of medication, both choice of agent and dose, and the need to balance against unwanted effects.
Use of alternative therapies^44^	Caring for patients who prefer to use alternative therapies; for example, traditional Chinese medicine, as opposed to prescribed medicines.
Use of Opioids^13,15,39,41,43–46^	Dilemmas surrounding the appropriate use of opioids, including under- and over-treatment, and patient and clinician opiophobia.

##### Confidentiality

3/13 included studies described challenges relating to confidentiality of information.^37,39,43^ All three studies reported participants’ belief in the importance of maintaining patient confidentiality, particularly in respect to loved ones.^43^

*‘During my assistance, I preserve patient privacy and maintain the confidentiality of the information I know about him [. . .] they are essential for the humanization of care’.* (Nurse, Brazil, p4926).^37^

##### Goals of care

All 13 studies reported ethical challenges relating to broader clinical considerations such as dilemmas concerning: withdrawing and withholding clinical interventions in the terminal phase^39–41,45^; patients’ preferred place of care and death^42,44^; overall therapeutic aims and strategies^44^ – particularly moving from disease-modifying to symptom-management-focused care; and treatment proportionality alongside assessments of futility.^13,15,37,38,40,43,45,47^ These challenges arose from practitioner interaction with multiple actors: patients, families, palliative care colleagues and external clinicians.^13,15,37,40,43,45,47^ A particular subset of these challenges related to treatment decisions or requests which were in conflict with expressed patient views.^45^ For example, in a German study the nurse participants viewed decision-making as to whether a patient should receive active life-prolonging therapy or more traditional palliative treatment as a ‘central ethical problem’.^43^

##### Mental capacity

In 5/13 studies, participants detailed ethical challenges concerned with the assessment of mental capacity and/or the role and choice of proxy decision makers for patients lacking decision-making capacity.^13,41,42,45,46^ For example, the authors of a USA study detailed challenges related to nurses identifying who was the correct person to be involved in decision-making and how to handle family conflict.^45^

##### Communicating with patients and families

Communication challenges were reported in 6/13 studies^13,15,39,42,43,45^ and included: inadequate quality of patient information (as perceived by practitioners), poor availability of staff to facilitate communication,^39^ poor inter-professional communication,^39^ differences in the cultural frames of reference within conversations,^43^ and managing conflicting information from and between multiple teams.^45^

*‘The provider offers unrealistic goals at the end of life, and continues treatment, often saying it is the family’s wishes, when the family does not have all of the information to make a realistic choice*.’ (Nurse, USA, p9)^45^

#### Working with families

In multiple studies, participants described ethical challenges derived from caring for the family and/or family involvement in patient care.^13,37,39–41,43,45–47^ Findings are in four sub-themes: care and support for the family; family as decision makers; genetics; and privacy.

##### Care and support of the family

In 3/13 studies^39,43,46^ participants reported challenges related to the care and support of the family. This included when adult patients do not want their illness discussed with their children^46^:

*‘A lot of times, parents won’t let us talk about [dying] with [children]. We can’t mention that word. . . But the kids are ready to talk about it. They need to talk about it. But a lot of times parents aren’t ready for that.’* (Social worker, USA, p956).^46^

Another challenge related to negotiating conflict between a family’s and patient’s wishes or support needs when it is not possible to satisfy both. Participants described how the patient must come first.^39^ A further challenge related to practicalities such as provision of overnight camp beds or food for families, and alongside this, which staff members’ responsibility it is to address these.^43^

##### Families as decision makers

Ethical challenges related to families’ role in decision-making were reported in 7/13 studies.^39–41,43,45–47^ Most participants detailed challenges arising from families requesting clinical interventions that health professionals thought were not in the patient’s best interests,^41,43,45–47^ or insisting on withholding diagnostic or prognostic information from the patient,^45^ particularly in situations where the patient lacked capacity to express their own wishes.^45–47^ Participants highlighted additional challenges of balancing supporting the family with responding to unrealistic demands, and prioritising the patient where disagreements occurred.^39,41,43,45–47^

*‘Patients are sometimes very passive, so the family decides for the patient. Or the patient agrees with the family just to please them. Our priority is the patient but we have to deal with the family also. If we get to the point they don’t agree, it’s the patient first.’* (Nurse, Canada, p1629)^39^

##### Genetics

In one study participants described the challenge of how to advise and support patients and families regarding genetic testing for conditions with an inheritable component.^14^

##### Privacy

In two studies^39,43^ participants reported concerns regarding the potential lack of privacy when families are involved in the care of patients.

*‘Maybe you don’t want the loved ones to be present in all situation [i.e. in caring for the patient’s intimate hygiene] . . . he also took pictures of some wounds . . .’* (Nurse, Sweden, p145)^43^

#### Engaging with institutional structures and values

Data in this theme related to institution-level decision-making or context, covering three sub-themes: conflict between healthcare professionals; conflict with institutional policies; and institutional resource allocation.

##### Conflict with institutional policies

Ethical challenges were experienced engaging with institutional policies that impacted on patient care, as found in 5/13 studies.^13,15,43,46,47^ Examples included: institutional policies prohibiting euthanasia in a jurisdiction where it is permitted^13^; medication availability and gaps in formularies^47^; gaps in insurance coverage preventing optimal management^47^; and routine do-not-attempt-resuscitation orders rather than individual decision-making.^43^

*‘And sometimes, I get resistance from the company that I work for because it (the medication) is not in the formulary. So the ethic part comes in: Who am I supposed to be taking care of? The patient? Or the bottom line of my company that I work for? And it’s very difficult. And there have been many times when I’ve been so vocal about it that I’ve actually gotten in trouble from the company, not from my families.’* (Nurse, USA, p13)^47^

##### Institutional resource allocation

Participants in 2/13 studies^39,44^ reported challenges related to institutional resource allocation, including: risks from delays in admissions due to lack of resources and staff availability,^39^ pressure to discharge due to policies limiting lengths of stay,^44^ and deficiencies in care quality due to perceived understaffing.^39,44^

‘*There are many patients here who just want to go home to die, but they cannot get the resources they need, so they are stuck here for their last days. They are frustrated about being here and they have trouble feeling dignified with all that is going on around them’* (Psychologist, Canada, p1629).^39^

##### Conflict between healthcare professionals

8/13 studies^15,39–41,43,45–47^ described the challenges of managing conflicting views between individual palliative care team members,^15,40,41,46^ other members of the multidisciplinary team,^46^ and other clinical specialties or disciplines.^39,43,47^ Conflicts were often described in terms of different individuals or professions prioritising different ethical principles.^46^

*‘After a discharge had been developed and approved by the primary care provider and patient/family, a specialist walking into the room and told the patient that he preferred another plan and offered dialysis’* (Nurse, USA, p9)^45^

#### Navigating societal expectations and values

Ethical challenges within this theme relate to broader societal or cultural values, legal and regulatory frameworks, and political landscapes that impact on day-to-day work.

##### Assisted dying

Participants in 6/13 studies^13,15,39–41,46^ described ethical challenges related to how to handle patients’ requests for assisted dying.^13,15,40,46^ In a Dutch study, staff reported that hospices’ opposition to euthanasia was a challenge, as euthanasia was available in other care settings.^13^ Institutions not performing assisted dying also was described as helping protect SPCPs from an ethical dilemma:

*‘You know, there’s a lot of people who . . . think [assisted suicide] is perfectly ﬁne . . . personally I don’t agree with [assisted suicide]. So, to me, that presents a dilemma. I mean, I’m off the hook because, of course, we don’t do that. . .’* (Social Worker, USA, p963)^46^

Participants in one German study were concerned about when opiate prescribing might be considered to overlap with ‘active assisted dying’.^41^

##### Conflict with wider societal rules, regulations or laws

This sub-theme arose in one study,^46^ in which practitioners described a conflict of duties for the healthcare professional when a patient’s autonomous choice conflicted with what is permitted under the licencing system of healthcare providers, or the law more generally.

‘*Any place [other than the hospice residence], we might be able to let a person do [whatever they want] but, because we’re licensed and we have under law assumed responsibility for the care and safety of these people, our responsibility has to supersede what the patient really wants to do.’* (Social worker, USA, p960)^46^

##### Access to specialist palliative care services

Lack of equity of access to palliative care services created ethical challenges for participants in one study.^42^

*‘the fairness of the system . . . many patients simply can’t afford being cared for by a specialized palliative care team such as ours because they don’t have the money to pay for it.’* (Nurse, Portugal, p726)^42^

##### Cultural and spiritual considerations

3/13 studies^15,39,43^ identified ethical challenges related to culture and spirituality. In a Swedish nursing study participants described these challenges as relating to a lack of mutual frames of reference or language which could affect the staff/patient relationship.^43^ The other two study reports did not provide details of the particular nature of the challenges.

##### Suicide

In two studies participants described challenges when patients reported suicidal ideation and planning.^15,46^

*‘I have had patients that have told me that they have a suicide plan, and my response is [to say] “Stop talking unless you want me to ﬁle whatever I have to ﬁle and make you stop.”’* (Social worker, USA, p963)^46^

#### Philosophy of palliative care

Challenges in this theme, which arose in 5/13 studies, related to practitioners grappling with the principles and aims of specialist palliative care.^13,38,40,41,43^ While these principles were not described in detail, participants described concerns about whether planned activities of care (such as life-prolonging treatment)^43^ were appropriate in palliative care.^41,43^

*‘Is it simply against the guiding principles of palliative care if I don’t only give him liquids now, but solids too?’* (Nurse, Germany, p326)^41^

*‘. . .but when you come in the morning and there you find, like, 10 bowls of blood samples . . . what is this. . . is this the ER? We want it to be a palliative care ward.’* (Nurse, Sweden, p144)^43^

Nurses in a Brazilian study expressed views about the remit and goals of palliative care that were notably different to the mainstream understanding of the field, for example:

*‘The patient does not need to know that he/she has a terminal illness, does not need to know that he/she has only a few days to live, does not need to know that there is no cure for his/her illness, does not need to know any of this.’* (Nurse, Brazil, p19)^38^

A broader ethical challenge in this sub-theme relates to the position specialist palliative care should take in relation to euthanasia and/or physician assisted suicide. Participants in Germany felt that opposition to these practices is important to maintain patient dignity, perceived as a key focus of palliative care.^40^ Dutch participants described a challenge occurring when patients make a request for euthanasia in the hospice as it is available in other care settings but not there.^13^ No study contained data supporting euthanasia within palliative care.

## Discussion

### Main findings

This review is the first systematic synthesis of the ethical challenges that specialist palliative care practitioners report encountering in their everyday clinical practice. We identified 13 studies from nine countries, and a wide range of ethical challenges across six main themes with 23 sub-themes. To the authors’ knowledge there are no similar systematic reviews in palliative care or other clinical fields, and therefore direct comparison is not possible.

Our findings have important implications for palliative care education. As educational curricula are not often publicly available, we examined the contents of specialty textbooks to understand the ethical challenges usually covered in palliative care ethics teaching. The breadth of the challenges we identified is striking: our findings are broader than those contained within the ethics chapters or sections in the ‘ethics’ sections of major palliative care textbooks (see Supplemental File 3 for a list of chapter titles).^51–55^ This reflects the findings of Hermsen and ten Have’s^13,14^ project examining this discordance. All topics in these textbooks are represented in the synthesis but not vice versa. The breadth of topics is also broader than the United Kingdom specialist palliative medicine physician training curricula.^56^

Hermsen and ten Have were the only researchers to use a bioethicist to observe workplace challenges. Although we have only included in the review the 31 challenges that were reported by practitioners within a hospice setting, across their full study they identified a total of 35 challenges across five research settings, far more than any other of the studies included in this review.^13^ It may be that the bioethicist observer identified scenarios as containing ethical challenges where a clinical practitioner might not have. This raises the possibility that not all of these challenges impact clinical care, or alternatively, that practitioners lack the training to recognise the full range of ethical challenges in the workplace. If ethical challenges are observer dependent, relying on practitioners’ viewpoints alone means certain challenges will be lost. As a consequence, there may be ethical challenges that are important to patients and carers but are missed by practitioners. A study of patients and carers in a single palliative care service in the UK illustrates this concern, describing the ethics of hope as a major finding, which is missing from these review findings.^57^ Further research is needed that focusses on patient and carer experiences of ethical challenges and triangulates the perspectives of specialist palliative care practitioners, ethicists, patients and carers.

We found ethical issues related to the philosophy of palliative care and whether certain clinical activities were appropriate in palliative care. While this is perhaps not surprising in a relatively young field, these findings reiterate how the demarcation and definition of specialist palliative care can differ between settings/contexts and over time. This is reflected in ongoing debates surrounding the definitions of palliative care^58–63^ and the shift to integrate palliative care alongside curative treatment.^58^

That participants expressed concern that opioids may shorten life in larger doses is also interesting. There is good evidence that appropriately titrated opioids do not shorten life.^64–66^ This finding highlights the ongoing challenge of promoting safe prescribing and the safety of opioids more generally, among colleagues, patients, and the public more broadly.

None of the studies we identified were undertaken in a low or low/middle-income setting. 10/13 were undertaken in high income countries,^13,39–41,43–47^ and 3/13 in two upper-middle-income countries, Brazil,^37,38^ and Mexico.^15^ This represents a significant evidence gap as 6.38 billion people live in low or low-middle-income countries^67^ and there may be specific ethical challenges that practitioners caring for patients in these settings face that are not represented within our findings. This concern is supported by the findings of a recent non-systematic review on the experiences of patients from non-Western and minority cultural backgrounds when using hospice and palliative care services, which highlighted multiple findings with clear normative elements that were prominent in low-income settings.^68^ Cultural differences across geographical locations have also been shown to impact on physicians’ experiences of ethical challenges.^69^ Although we did identify some challenges related to cultural and spiritual aspects of care, it is perhaps unexpected that these topics were so infrequently represented.

The findings of this review have clear implications for ethics support services and palliative care training. We identified challenges that were predominantly related to specific scenarios or contexts rather than the general application of broader ethical principle frameworks. This finding of a context-focus is in line with systematic reviews examining ethics within dementia care^70,71^ and nursing,^72^ as well as individual studies in general practice,^11^ community pharmacy,^73^ Canadian hospitals,^74^ renal medicine,^12^ pain medicine^10^ and generalist end of life care.^75–79^ The focus on detail has been termed ‘microethics’, and proponents argue that this is the level at which most ethically challenging decision-making occurs.^80^ This contextual structure is important when considering how to support and manage ethical decision-making in the healthcare environment. Practitioners are often taught to analyse case-based scenarios using principle-based frameworks such as Beauchamp & Childress’ ‘Four Principles’ approach.^80,81^ However, our findings support the use of alternative approaches which explicitly consider context-based facts, such as the Seedhouse grid,^82^ or the Four Quadrants approach.^83^ In addition, outcome-based ethical assessment frameworks such as utilitarianism, virtue ethics, and ethics of care,^84–87^ more easily incorporate individual context than do a priori, rule-based, deontological frameworks.

Finally, only a relatively small proportion of the challenges reported by participants related directly to clinical decision-making about individual patients. Challenges were located across the care environment, from the bedside through institutional and societal values to national-level policy. Those wishing to engage with ethics within palliative care when aiming to improve care at the bedside must therefore acknowledge the impact of these multiple levels. This has relevance to palliative care ethics education, which often relies on patient case-based teaching.^80,88^ Care must be taken in the writing of cases to include themes that are located away from the bedside.

## Strength and limitations

Strengths in the design of the review include its systematic approach; lack of language, geographical, or date restrictions in the search protocol; the inclusion of LILACS to better capture non-English language research; and quality assessment of both included studies and review findings. However, study design decisions are also associated with potential limitations. First, the search strategy used methodological filters. Pilot filtered searches were evaluated for study loss using pre-identified sentinel studies; all were returned by the search strategy. However, it remains possible that relevant studies were missed due to misclassification in the registry or novel methodology. Second, searching in English only might also mean that relevant articles not published in English and indexed only in non-English databases were missed.

Third, the contributing studies often did not contain detailed description of the nature of the ethical challenges reported. Our review is similar to other broader healthcare ethical challenge reviews in that in depth analysis of every challenge was not possible.^89^ Two papers with lower MMAT scores reported a small number of ethical challenges with insufficient explanatory context to accurately include them in the synthesis. These challenges were: existential suffering care,^13^ unbearable suffering,^13^ motivation,^13^ paediatric palliative care,^13^ patients feeling a burden to their families,^39^ quality of care,^13^ quality of life,^13^ research with terminally ill patients,^13^ responsibility,^13^ and role as a researcher.^13^

Finally, quality assessment of qualitative research and its outputs is a contested area, with multiple tools available and poor correlation between methods.^90^ The MMAT contains fewer criteria to assess study quality than methodology-specific tools and may lead to an incorrect over- or under-assessment of a study’s inherent bias. However, we did not exclude studies based on their MMAT assessments and believe the ability to directly compare studies of differing methodologies was useful. The GRADE CERQual approach helps to systematise the assessment of the findings of the review but is underpinned by researcher judgement, allowing for possible mis-categorisation.

## What this study adds?

This is, to our knowledge, the first review to systematically detail specialist palliative care practitioner-reported ethical challenges and has important implications for palliative care and ethics training. The identified ethical challenges are far broader than those included in current major textbooks in the field. These challenges are located at diverse levels, from the bedside up to national policy. We found no data from low and middle-income settings where the majority of the world’s population live and die. Finally, this review, through the breadth of data synthesised, demonstrates the utility of robust methodologies within empirical bioethics. That the review identified ethical challenges that are not included in the major textbooks reinforces the need for this approach alongside theoretical aspects of bioethics, if the aim is more ethically-informed clinical care.

Further research is needed to explore patients and carers’ perspectives, the nature of the individual challenges identified in this review, and how these may vary across settings and countries.

## Supplemental Material

sj-pdf-1-pmj-10.1177_0269216320974277 – Supplemental material for Real-world ethics in palliative care: A systematic review of the ethical challenges reported by specialist palliative care practitioners in their clinical practice Click here for additional data file. Supplemental material, sj-pdf-1-pmj-10.1177_0269216320974277 for Real-world ethics in palliative care: A systematic review of the ethical challenges reported by specialist palliative care practitioners in their clinical practice by Guy Schofield, Mariana Dittborn, Richard Huxtable, Emer Brangan and Lucy Ellen Selman in Palliative Medicine

sj-pdf-2-pmj-10.1177_0269216320974277 – Supplemental material for Real-world ethics in palliative care: A systematic review of the ethical challenges reported by specialist palliative care practitioners in their clinical practice Click here for additional data file. Supplemental material, sj-pdf-2-pmj-10.1177_0269216320974277 for Real-world ethics in palliative care: A systematic review of the ethical challenges reported by specialist palliative care practitioners in their clinical practice by Guy Schofield, Mariana Dittborn, Richard Huxtable, Emer Brangan and Lucy Ellen Selman in Palliative Medicine

sj-docx-3-pmj-10.1177_0269216320974277 – Supplemental material for Real-world ethics in palliative care: A systematic review of the ethical challenges reported by specialist palliative care practitioners in their clinical practice Click here for additional data file. Supplemental material, sj-docx-3-pmj-10.1177_0269216320974277 for Real-world ethics in palliative care: A systematic review of the ethical challenges reported by specialist palliative care practitioners in their clinical practice by Guy Schofield, Mariana Dittborn, Richard Huxtable, Emer Brangan and Lucy Ellen Selman in Palliative Medicine
