# Supplementary material for: Real-world ethics in palliative care: A systematic review of the ethical challenges reported by specialist palliative care practitioners in their clinical practice
Source: Palliat Med. 2020 Dec 10;35(2):315–34. doi: 10.1177/0269216320974277 (PMC7897798; doi:10.1177/0269216320974277)
Supplement: sj-pdf-1-pmj-10.1177_0269216320974277 – Supplemental material for Real-world ethics in palliative care: A systematic review of the ethical challenges reported by specialist palliative care practitioners in their clinical practice [file sj-pdf-1-pmj-10.1177_0269216320974277.pdf]

Supplementary File 1: Methodological Filter Use

Database	Qualitative Filter	Survey Filter
Medline	http://libguides.sph.uth.tmc.edu/search_filters/ovid_medline_filters	Bespoke
Embase	http://libguides.sph.uth.tmc.edu/search_filters/ovid_medline_filters<br>again used with an OR with:<br>https://hiru.mcmaster.ca/hiru/HIRU_Hedges_EMBASE_Strategies.aspx#Qualitative	Bespoke
PsycINFO	https://hiru.mcmaster.ca/hiru/HIRU_Hedges_PsycINFO_Strategies.aspx	Bespoke
CINAHL	High sensitivity filter from<br>https://guides.nyu.edu/c.php?g=277034&p=1846828<br>Peer reviewed: [1]	Bespoke
Philosophers Index	Bespoke. No peer reviewed strategies available.	Bespoke
LILACS	Not used as small number of results	Not used
Web of Science	Bespoke. No peer reviewed strategies available.	Bespoke

The Medline bespoke survey filter is as follows:

1 exp "Surveys and Questionnaires"/

2 survey*.mp.

3 question*.mp.

4 or/1-3

5 ((("semi-structured" or semistructured or unstructured or informal or "in-depth" or indepth or "face-to-face" or structured or guide) adj3 (interview* or discussion* or questionnaire*)) or (focus group* or qualitative or ethnograph* or fieldwork or "field work" or "key informant")).ti,ab. or interviews as topic/ or focus groups/ or narration/ or qualitative research/

6 4 or 5

References:

1. Wilczynski NL, Marks S, Haynes RB. Search Strategies for Identifying Qualitative Studies in CINAHL. *Qual Health Res* 2007; 17: 705–710.
