# Supplementary material for: Real-world ethics in palliative care: A systematic review of the ethical challenges reported by specialist palliative care practitioners in their clinical practice
Source: Palliat Med. 2020 Dec 10;35(2):315–34. doi: 10.1177/0269216320974277 (PMC7897798; doi:10.1177/0269216320974277)
Supplement: sj-pdf-2-pmj-10.1177_0269216320974277 – Supplemental material for Real-world ethics in palliative care: A systematic review of the ethical challenges reported by specialist palliative care practitioners in their clinical practice [file sj-pdf-2-pmj-10.1177_0269216320974277.pdf]

Supplementary file 2: CERQUal Evidence Profile (Full)

Summary of Review finding	Studies contributing to the review finding	Methodological limitations	Coherence	Adequacy	Relevance. Alternative criterion – see explanatory text	CERQual assessment of confidence in the evidence	Rationale for CERQual assessment
1. *Application of Ethical Principles.* Ethical challenges that relate to practitioners applying bioethical principles to clinical situations							
1.1 *Autonomy*. Challenges related to the bioethical principle of autonomy	(1–8)	Minor concerns.<br><br>8/13 studies contributed to this finding. No or minor concerns were present for 4 studies, 3 studies were graded as moderate and 1 for serious concerns. This is a descriptive review and despite the flaw in methodology, or lack of data preventing full assessment, the results in this finding were represented, and were similar in concept, in both the higher and lower quality studies.	No concerns	Minor concerns.<br><br>This review finding is found within the majority of included studies. This is a descriptive review and all studies detailed enough information to interpret and extract this finding to an adequate level.	Minor concerns.<br><br>The included studies were undertaken in high income (5/8) or upper middle income (3/8, Mexico, Brazil) settings. No studies examined this issue in lower middle- or lower-income settings. The concepts within the challenges were consistent across the included studies.	High confidence.	This review finding was present in 8/13 included studies. Despite methodological concerns the data in this finding was consistent across the included studies. The authors have high confidence challenges related to autonomy feature in every day palliative care practice.
1.2 *Dignity*. Challenges that engage any variant of the concept of dignity	(1,4,6,7)	Minor concerns.<br><br>4/13 included studies contributed to this review finding, of which 3 studies were rated as no or minor concerns and one as moderate concern.	Moderate concerns.<br><br>The included studies describe divergent possible content of the concept of 'dignity'. The concept of dignity also overlapped with other themes.	Moderate Concerns.<br><br>The content of the included studies was relatively thin and all coded sections reported dignity in the context of an accompanying concept: suffering (1 study), autonomy (3 studies), absence of provision of euthanasia (2 studies). Dignity was not conceptualised withing itself in any included study.	Minor concerns<br><br>The 4 included studies came from Brazil, Canada, Germany and the USA and so may not represent the global view the review aims to address.	High confidence.	Despite a rating of moderate concerns for coherence, and adequacy, this is a broad descriptive review and the authors have confidence that specialist palliative care practitioner's engage with dignity within their everyday ethical challenges. The nature of dignity is highly contested in bioethics and the review findings reflect this.

Supplementary file 2: CERQUal Evidence Profile (Full)

1.3 *Truth Telling*. Participants reported ethical challenges related to the ethical principle of truth-telling. This focuses around whether it was appropriate to discuss both the terminal nature of a diagnosis and/or prognosis to both the patient and to family members. It also covered whether it may be appropriate to withhold information from patients, either because of clinician beliefs regarding harms, or the wish of the families.	(2–5,7,9–13)	Minor concerns.<br><br>10/13 included studies contributed to this finding. Five studies were assessed as no or minor concerns, four further studies as moderate concerns and one as serious concerns.	Minor concerns.<br><br>The contributing studies all report similar concepts within this finding. There are differences in the approach to truth telling between studies, but all report it as a challenge that the contributing participants faced.	Minor concerns.<br><br>The multiple contributing studies contain varying degrees of details regarding this challenge. However, they are sufficient for the descriptive aim of this review.	Minor concerns<br><br>The included studies are drawn from across the range of settings of the included studies and include all professional backgrounds. There is a lack of research situated in low and low-middle income settings to contribute to this review.	High confidence.	This finding is contributed to by most of the studies included in the review. There is a high level of coherence between the individual study findings. The authors are confident that ethical challenges related to truth telling form part of the network of challenges faced by practitioner in day to day clinical practice.
1.4 *Doctrine of Double Effect.* Challenges that relate to scenarios where the doctrine of double of effect is thought to have been enacted, usually around the prescription and effects of opioids.	(2,5,8,10)	Moderate concerns.<br><br>This finding was reported in 4/13 studies. Two were rated as having moderate concerns and one as having serious concerns.	Moderate concerns.<br><br>Only two of the four studies had detailed descriptions of the content of the challenges. There is therefore a lack of data to facilitate a detailed assessment for coherence.	Moderate concerns.<br><br>Only two reporting studies the doctrine of double effect contained details of the challenges descriptive enough to understand the concept. The other two studies did not contain enough data to adequately cross-reference the content.	Moderate concerns.<br><br>The included studies were from Brazil, Mexico and Western Europe and so may not necessarily represent the global viewpoint. The paucity of primary data exacerbates this.	Moderate confidence.	The authors are moderately confident that SPCPs felt that they experienced ethical challenges engaging with the doctrine of double effect as described in the contributing studies. There is a lack of detailed description in the primary data and the locations of the source studies, in both geography and in time, may suggest a variance in how widely this is experienced.
1.5 *Equity in care*. Ethical challenges that engage with promoting equity in the delivery of palliative care across multiple patients.	(1,5,13)	Moderate concerns.<br><br>This finding was derived from 3/13 included studies. One was assessed as no or minor concerns and	Serious concerns.<br><br>There is limited data available from all three studies. The content of this finding differs significantly	Serious concerns.<br><br>Although the finding is based on three studies, there are two divergent topics, and both are ‘thin’.	Moderate concerns<br><br>The data is derived from three studies which may contain specific contextual factors that influence	Moderate confidence.	The authors assessed that whilst there are multiple areas of concern for this finding, it must be viewed in the context of this review being

Supplementary file 2: CERQUal Evidence Profile (Full)

		two with moderate concerns. There are specific concerns about rigour of analysis for this finding.	between the included studies and there is limited coherence between the two areas covered.		the nature of the findings and may not be present in other geographical locations.		very descriptive in nature. Detailed understanding of the concept is not required for the review to meet its aims. Therefore, the authors have moderate confidence that issues regarding equity in care are experienced in clinical practice amongst specialist palliative care practitioners.
1.6 *Fidelity*. Ethical challenges exist that relate to the professional ethics value of fidelity, This relates to the value of adhering to the professions core values.	(12)	No concerns.<br><br>This finding was reported in a single included study that was rated as high quality by the MMAT 2018 tool and assessed as having no methodological concerns.	No concerns.<br><br>The single study contained only two references to this ethical challenge that are congruous with each other. The finding directly represents the findings of the contributing study.	Significant concerns.<br><br>This finding was drawn from a single study that presented thin data on the topic.	Moderate concerns<br><br>The single study this finding was derived from had a narrow pool of participants – 6 hospice nurses. The narrow setting and single professional background limit the ability of the research to assess how this may relate to the larger field of palliative care more broadly.	Moderate confidence.	The authors felt that although this finding was derived from a high quality recently published study the paucity of primary data and the narrow range of participants (nurse only) allowed, at best, for only moderate confidence in this finding.
2. *Delivering Clinical Care.* Ethical challenges embedded in clinical decision-making and bedside care							

Supplementary file 2: CERQUal Evidence Profile (Full)

2.1 *Clinical care and decision-making*. Ethical challenges that relate to the provision of specific clinical interventions rather than more general goal-based decision making. There is some unavoidable overlap with goals of care (2.4).	(2–11,13)	Minor concerns.<br><br>11/13 included studies contributed to this finding. 6/13 studies were assessed as no or minor concerns, 4/13 as moderate and 1 as serious concern.	Minor concerns.<br><br>The contributing studies contain a wide variety of challenges that contribute to this finding. Presenting this as a collection of sub-findings allows for closer coherence with the contributing studies.	Minor concerns.<br><br>The specific nature of the clinical interventions described in the contributing studies facilitated understanding of the concepts with thinner detail than would be needed for broader bioethical concepts.	Minor concerns.<br><br>The contributing studies were undertaken in all the geographical areas represented in the overall included studies.	High confidence.	The authors assess this finding as high confidence. The variety of challenges grouped that made up the sub findings were described across multiple contributing studies, represented all geographical and income settings of the included studies and represented views from a broad range of participant professional backgrounds.
2.2 *Confidentiality.* Ethical challenges relating to clinical confidentiality.	(1,7,13)	Moderate concerns.<br><br>3/13 included studies contributed to this finding. Two were assessed as no/minor concerns and two as moderate concerns.	Minor concerns.<br><br>The finding is closely related to the content of the contributing studies. The concept of confidentiality is arguably less nuanced than some of those in other findings and all primary data corresponds to the concept and its importance.	Moderate concerns.<br><br>The contributing studies are very thin on this topic. That it exists as an area for challenges in clear, but this assessment area would be rate more highly with richer data.	Minor concerns.<br><br>The contributing studies were performed in high- and upper-income contexts, North American and European, and across a range of participant professional backgrounds. No additional concerns are raised here other than those raised above relating to lo and low-middle income settings.	High confidence.	Despite the range of moderate concerns, the authors have assessed a high confidence in this finding. The concept of confidentiality is usually well understood amongst healthcare professionals and in the literature, and therefore can be understood with thinner data than might be required for other findings.
2.3 *Goals of care.* This focused decision-making on broader aspect of broader aspects of clinical care, as compared to the more focused medical sub-	(1–13)	Minor concerns.<br><br>All included studies contributed to this finding. 7 studies were assessed as no or	Minor concerns.<br><br>All included studies contributed to this finding and the majority of	Minor concerns.<br><br>There is a broad range of descriptive detail across the contributing studies.	Minor concerns.<br><br>All included studies contributed to this review. The broader concerns of the	High confidence.	The authors assess having high confidence in this finding. The finding is contributed to by all included papers with a

Supplementary file 2: CERQUal Evidence Profile (Full)

theme that focuses on individual clinical interventions. Broader topics included dilemmas concerning, overall therapeutic aims and strategies, particularly around clinical decision-making moving from disease modifying to symptom management only, withdrawing and withholding, treatment proportionality and assessment of futility, and patient's preferred place of care and death.		minor concerns, 5 as moderate and 1 as serious concern.	contributing studies describe similar ethical challenges. There is one outlying study which contains the same headline challenges but suggests its participant focus on different aspects and diverge from more commonly held positions. The level of concern this generates is small as this is a descriptive review and not an explanatory one.	This satisfies the need of a descriptive review.	high/upper-middle income bias of these studies is present in this finding.		high level of coherence in the high-level nature of the challenges. Deeper concepts within the challenges do slightly differ in one Brazilian study but that goals of care is a challenge is not disputed.
2.4 *Mental capacity*. Participants reported ethical challenges were experienced relating to both the assessment of mental capacity and the role and choice of proxy decision makers should capacity be assessed as being impaired in a specific instance.	(3–5,8,11)	Moderate concerns.<br><br>5/13 included studies contributed to this finding. 3 studies were assessed as no or minor concerns and two were assessed as moderate concerns.	Minor concerns.<br><br>The contributing studies had a high level of coherence in relation to each other and therefore the content of this finding.	Minor concerns.<br><br>The richness of the content was adequate for a descriptive review.	Minor concerns.<br><br>The contributing studies contained a range of healthcare professional backgrounds and matched the geographical range of the total set of included studies.	High confidence.	The authors assess this finding as high confidence. The contributing studies represented the participants across all included studies as a whole in both range of professional backgrounds and geographical context. The contributing studies had good levels of coherence between themselves allowing coherence with the review finding.
2.5 *Communication with patients and their families.* Reported challenges included; perceived quality of information given, including managing conflicting information from multiple teams, poor availability of staff to facilitate communication, communication between	(3,5,7,10,11,13)	Minor concerns.<br><br>6/13 include studies contributed to this finding. 3 studies were assessed as no or minor concerns and 3 were assessed have moderate concerns.	Moderate concerns.<br><br>The studies that contribute to this review finding report on multiple aspects of difficulties in communication. The limited data from each study limits understanding of the	Moderate concerns.<br><br>The contributing studies have relatively thin data and contain a wider range of sub-topics than in other review findings. Therefore, the authors asses this as a moderate concerns.	Minor concern.<br><br>The contributing studies represent all the geographical contexts of the broader group of included studies.	Moderate confidence.	The authors rate this finding as only moderate confidence as, although it is clear there is an area of challenges described that is distinct from other findings areas (such as truth telling above), there is a thinness in the data

professionals, and differences in the cultural frames of reference within conversations.			issue raised and collating these topics risks further distance being created between the finding ant the individual study authors findings.				regarding the content of this finding. It may be that richer data described a different range of content for this finding as some contributing primary study data may have been re-coded to other findings, altering the content of this one.
3. *Working with Families.* Multiple ethical challenges are experienced that are derived from working alongside families as they support their relatives, from care of the family members themselves, or as a whole.							
3.1 *Family as decision makers.* Challenges arising from when families requested clinical interventions that the health professionals thought were not in the patient's best interests or insisted on the withholding of diagnostic or prognostic information from the patient. These were particularly pronounced in situations where the patient lacked capacity to express their own wishes.	(3,4,6–8,12,13)	Minor concerns.<br><br>7/13 included studies contributed to this finding. Six studies were assessed as no or minor concerns and one as moderate.	Minor concerns.<br><br>The multiple contributed studies contained similar conceptual details on the nature of this grouping of challenges and this was able to be reflected in the finding.	Moderate concerns.<br><br>The contributing studies vary in the richness of the data relating to this finding, but the majority comes from higher quality studies.	Minor concerns.<br><br>The contributing studies represent all healthcare professional background but are limited to high income research settings only.	High confidence.	The authors have high confidence in this finding. The coherence between the contributing studies, and their high methodological assessment, is supportive of the finding being one of the ethical challenge areas that are experienced by palliative care practitioners.
3.2 *Care and support for the family.* Challenges related to the care/support of patients' families and children. This finding focused on the care of children, particularly where the professionals view on the best way of the supporting the child differed from the patient or family member, and separately support for family on the ward in terms of bedding/food etc.	(4,7,13)	Minor concerns.<br><br>This finding was contributed to by 3/13 included studies. All were assessed as having minor concerns. These were predominantly due to lack of reflexivity and data saturation.	Minor concerns.<br><br>This finding collates two separate areas of family care, although there is coherence between the contributing studies. This coherence was therefore able to be reflected in the finding.	Moderate concerns.<br><br>The contributing studies provides enough detail to summarise the primary data into this finding but not enough to detail the deeper conceptual content.	Moderate concerns.<br><br>This finding was derived from three studies. Two studies enrolled a single professional group – nurses and nursing assistants, and social workers respectively. The third study interview more broadly but was published in 2003. Therefore, there may	Moderate confidence.	The authors rate this finding as moderate confidence. Derivation from high quality studies supports its inclusion at the finding level, but the thinness of the data and the disparity between the studies' results that, whilst there is confidence this is a challenge area, the exact nature of these challenges is not

Supplementary file 2: CERQUal Evidence Profile (Full)

					be issues of relevance with regards other health professional groups and as before, to other geographical settings.		reliably discernible from the primary data.
3.3 *Genetics.* Practitioners report challenges when supporting or advising patients and their families about genetic elements of conditions that are potentially hereditable.	(5)	Moderate concerns.<br><br>The single contributing study is rated as moderate concern relating to sample, data collection, and reflexivity. It was a mixed methods study and the only included study to incorporate ethnography by a bioethicist.	No concerns.<br><br>As there was only one contributing study the finding is coherent with the primary data.	Serious concerns.<br><br>The data that underpins this finding is thin. It lacks the depth for a detailed understanding of the challenge.	Serious concerns.<br><br>The contributing study was published in 2003. The science and practice of genetics has advanced considerably in this time and so it may not reflect the current format of challenges in this area.	Moderate confidence.	This finding is rated as moderate confidence. The authors have moderate confidence that there is a collection of challenges related to genetics in the daily practice of specialist palliative care. However, the exact nature of this challenge cannot be determined from the data contained within the contributing study.
3.4 *Privacy*. Challenges around families being present for care episodes that the practitioners felt was not appropriate.	(7,13)	Minor concerns.<br><br>2/13 included studies contributed to this finding. Both were assessed as minor concerns. These focused around sampling and saturation.	Minor concerns.<br><br>The two contributing studies had coherence between their findings, and this was reflected in the make-up of this review finding.	Moderate concern.<br><br>Both studies contained limited data to help understand the nature of this finding.	Minor concerns.<br><br>The two contributing studies were both set in high-income settings but enrolled a wide range of participants. They were published 12 years apart and so this may support this as an ongoing area of challenges.	Moderate confidence.	This finding is assessed as moderate confidence. The studies that contribute to it scored well on methodology, and the finding is coherent. There is however limited detailed primary data and so again as this is a very descriptive review the level of confidence is higher than it might have been should an explanatory power be required.
4. *Engaging with Institutional Structures and Values.* Ethical challenges that engage with values held by other professionals, professional groups or healthcare institutions.							
4.1 *Conflict with institutional policy.* In this finding ethical challenges were experiences when engaging with institutional	(4,5,10,12,13)	Minor concerns.<br><br>This finding was contributed to by 5/13 included studies.	Moderate concerns.<br><br>Whilst all contributing studies identified institutional policy	Moderate concerns.<br><br>The multiple variations in the details of the individual examples	Moderate concerns.<br><br>The contributing studies did not fully represent the settings	High confidence.	This finding is assessed as high confidence. The contributing studies are clear in identifying that

Supplementary file 2: CERQUal Evidence Profile (Full)

and organisational policies that impacted on the participants care of the patients.		Three studies were rated as no or minor concerns and two were rated as moderate concerns.	from care providers or insurance providers as the source of the ethical challenges, the details of the challenges varied significantly between contributing studies.	that made up this finding were underpinned by thin data.	captured by the full set of included studies. Some aspects of this finding will likely only be applicable in limited geographical settings, for example jurisdictions where euthanasia is an option. These are limited at the time of writing but may increase over time.		engaging with institutional policies create ethical challenges in the care of individual patients. The content of the challenges varies between studies, but the overarching concept is rich enough for a descriptive review and for an assessment of high confidence.
4.2 *Institutional resource allocation.* This finding focused around internal resource allocation. This is variously manifested; risks from delays in admissions due to lack of resources and staff availability, pressure in institutions for limited lengths of stay and deficiencies in the quality of care resulting from perceived understaffing.	(7,9)	Moderate concerns.<br><br>2/13 included studies contributed to this finding. One was assessed as having minor methodological concerns, and one as moderate.	Minor concerns<br><br>There is a good level coherence between the coded sections in each of the included studies, and with the finding overall.	Minor concerns.<br><br>Contributing studies contain a relatively high level of detail in the descriptions of the nature and effects of challenges in this finding.	Minor concerns.<br><br>The included studies contain similar ethical content despite the range in geographical location, and years of publication. The persistence of these themes increases the likelihood of relevance. One included study also included the views of patients and their families and the data from them support this as a source of ethical challenges.	High confidence.	The authors judge this finding has having high confidence. The coherence between the contributing studies and the range of the contributing studies supports the finding that resource allocation creates ethical challenges for individual healthcare practitioners.
4.3 *Conflict between healthcare staff*. Ethical challenges that conflict between individual palliative care team members, conflict between members of the multidisciplinary team that the participants felt was related to differing professions prioritising different ethical principles, and conflict with other	(3,4,6–8,10,12,13)	Minor concerns<br><br>8/13 included studies contributed to this finding. Six studies were rated as no or minor concerns, and two studies were rated as moderate concerns.	Minor concerns.<br><br>There contributing studies each detailed the interaction between staff as a source of challenges, but the deeper content varied significantly in both detail but also more general conceptual content. Despite this	Moderate concerns.<br><br>All but one of the contributing studies contained very limited detail on the nature of the challenges, even given the lower requirements for a descriptive review.	Minor concerns.<br><br>The contributing studies containing data from a representative rage of professional backgrounds.	Moderate confidence.	The authors assess this finding as moderate confidence. The low level of coherence between the contributing studies in both the detail and more general content increases the risk that although challenges of this nature exist, this review may not

clinical specialties or disciplines.			variation the review finding was able to capture this in a level of detail appropriate for this descriptive review.				accurately capture their content.
5. *Navigating Societal Values and Expectations.* Ethical challenges that intersect with values in society more broadly, situated beyond the healthcare practitioner or healthcare institution.							
5.1 *Assisted Dying*. Ethical challenges related to how to handle patient's requests for assisted dying, rather than the more general question about whether it should be available as an option. This finding also explored participant concerns of the border between appropriate opioid prescribing and hastening death.	(4–8,10)	Minor concerns.<br><br>This finding was contributed to by 6/13 included studies. 4 studies were rated as no or minor concerns and 2 were rated as moderate concerns.	Minor concerns.<br><br>The contributing studies were congruous in their approach to this issue.	Minor concerns.<br><br>The richness of data on this finding was deeper than for most other findings in this review and sufficient for a descriptive review. The finding aspect related to the doctrine of double effect contained enough detail to understand the concept.	Minor concerns.<br><br>The contributing studies contained research from jurisdictions that allowed euthanasia and assisted dying and those that didn't. It represented high and upper-middle income settings and a full range of professional backgrounds.	High confidence.	The authors assess this finding as high confidence. The range of contributing studies, and level of agreement between them in the formulation of the challenge support this assessment.
5.2 *Conflict with wider societal rules, regulations or laws.* This finding pertains to a conflict of duties for the healthcare professional when a patient's autonomous choice conflicts with what is permitted under the licencing system of healthcare providers, or the law more generally.	(4)	No concerns.<br><br>One study contributed to this finding and it was assessed as being of high quality by the MMAT 2018 tool.	No concerns.<br><br>As this finding is based on a single contributing study which contained a clear explanation of the nature of the finding there are no concerns regarding coherence of the finding relating to the primary data.	Moderate concerns.<br><br>The contributing study was clear on the higher level nature of the challenge but data justifying this position was thin.	Moderate concerns.<br><br>The contributing study examined the ethical challenges experience by social workers in one Canadian province. This is not necessarily reflective of the wider specialist palliative care workforce or other global contexts.	Moderate confidence.	The authors assess this as moderate confidence. The finding derives from a single high-quality study. This rigour in method helps support the inclusion of the finding in this descriptive review. What is less certain is the exact nature of the challenges, and how this may change across professional groups and service locations.
5.3*Access to specialist palliative care*. Inequitable access to specialist palliative care services	(11)	Minor concerns.<br><br>The contributing study was rated as having	No concerns.	Serious concerns.<br><br>There is limited detail on the effects of this	Moderate concerns.<br><br>The included study setting was Portugal	Low/moderate confidence	The authors assess low/moderate confidence in this finding. Whilst the

leading to perceived harms to the affected individuals. This finding is tied to the bioethical concept of justice.		minor concerns regarding its methodology, particularly concerning the mixed methods design and quantitative sections. This finding is derived from the qualitative section of the contributing study.	This finding is directly derived from the contributing study	challenges on patients or staff.	and only recruited nurses and physicians, so there is a risk that important viewpoints are missing from this finding.		single contributing study is clear in locating the source of the challenge, it lacks enough detail to understand it in more detail that title only.
5.4 *Cultural and spiritual considerations.* Ethical challenges were identified that were based in a possible lack of mutual frames of reference, language, and cultural and spiritual practices regarding death and dying.	(7,10,13)	Moderate concerns.<br><br>3/13 included studies contributed to this finding.	Moderate concerns	Serious concerns.<br><br>The three contributing studies have very little detail on the context or concepts related to this finding. This thinness results in serious concerns about the adequacy of the content that forms this finding and this the nature of the finding itself.	Moderate concerns.	Low confidence.	The authors assess this finding as low confidence based on the data from the contributing primary studies. That ethical challenges may be based in cultural and spiritual aspects of care is clearer, but the specific description of this finding as derived from the thin underlying data is potentially unreliable.
5.5 *Suicide.* Ethical challenges that relate to how practitioners should respond to patient reports of suicidal ideation and planning.	(4,10)	Minor concerns.<br><br>2/13 included studies contributed to this finding. One was assessed as high quality and the second as having moderate methodological concerns.	Minor concerns.<br><br>There is limited data contributing to this finding, and the detailed data is from one study only. Therefore, the findings match this data closely, resulting in minor concerns for coherence.	Serious concerns.<br><br>The primary data for this finding comes from only two studies and is thin. There are serious concerns as to the adequacy of this finding.	Moderate concerns.<br><br>The primary data is derived predominantly from a high-quality study that by design, only recruited social workers in one region of Canada and therefore the relevance of this finding to other settings is not clear.	Low confidence.	The authors assess this finding as serious concerns. The thinness of the data that contribute to this finding, and the inclusion of only 2 studies results in low confidence in the content of the finding, as it is potentially likely that more detailed exploration of the topic would generate conflicting and contrasting data points.
6. *The Philosophy of Palliative Care.* Ethical challenges relating to how provision of particular approaches to care might be judged from differing viewpoints related to the nature and aims of palliative care.							

Supplementary file 2: CERQUal Evidence Profile (Full)

6.1 *Philosophy of palliative care*. Participants reported challenges related to the very nature of the aims of the field of specialist palliative care itself.	(2,5,6,8,13)	Moderate concerns.<br><br>5/13 included studies contributed to this finding. 3 were rated as no or minor concerns and two were considered as moderate concerns.	Serious concerns.<br><br>There were clear deviant case data in this finding that the relevant study authors identified as such. These were difficult to collate into a coherent finding with the other primary data.	Moderate concerns.<br><br>There is limited data on all aspects of this finding across all included studies which limits the authors ability to collate the primary findings. This review finding is a deeper concept than some of the others and so requires more detailed primary data, which is lacking.	Moderate concerns.<br><br>The contributing studies are limited to Europe and Brazil and may therefore not speak to experiences in other areas of the globe.	Moderate confidence.	The authors have moderate confidence in this finding. The deeper details are less clear but that there are challenges that are the result of more abstract debates about the nature and role of palliative care filtering down to the bedside seems clear.

Supplementary file 2: CERQUal Evidence Profile (Full)

1. Andrade CG de, Andrade MI de, Brito FM de, Costa ICP, Costa SFG da, Santos KFO dos. Cuidados paliativos e bioética: estudo com enfermeiros assistenciais Palliative care and bioethics: study with assistance nurses. Rev Pesqui Cuid É Fundam Online. 2016 Oct 4;8(4):4922.
2. Bezerra do Amaral J, Menezes M do R de, Martorell-Poveda MA, Cardoso Passos S. Ethic and bioethic dilemmas on palliative care for hospitalized elderly: nurses' experience. Cult Los Cuid Rev Enferm Humanidades. 2012;16(33):14–21.
3. Cheon J, Coyle N, Wiegand DL, Welsh S. Ethical Issues Experienced by Hospice and Palliative Nurses. J Hosp Palliat Nurs. 2015 Feb;17(1):7.
4. Dennis MK, Washington KT, Koenig TL. Ethical Dilemmas Faced by Hospice Social Workers. Soc Work Health Care. 2014 Nov 26;53(10):950–68.
5. Hermsen MA, Ten Have HAMJ. Moral Problems in Palliative Care Practice: A Qualitative Study. Med Health Care Philos Eur J. 2003;6(3):263–72.
6. Salloch S, Breitsameter C. Morality and Moral Conflicts in Hospice Care: Results of a Qualitative Interview Study. J Med Ethics J Inst Med Ethics. 2010;36(10):588–92.
7. Towers A, MacDonald N, Wallace E. Ethical issues in palliative care. Views of patients, families, and nonphysician staff. Can Fam Physician. 2003 Dec;49:1626–31.
8. Walker A, Breitsameter C. Ethical decision-making in hospice care. Nurs Ethics. 2015 May;22(3):321–30.
9. Chiu T-Y. Ethical dilemmas in palliative care: a study in Taiwan. J Med Ethics. 2000 Oct 1;26(5):353–7.
10. Guevara-López U, Altamirano-Bustamante MM, Viesca-Treviño C. New frontiers in the future of palliative care: real-world bioethical dilemmas and axiology of clinical practice. BMC Med Ethics [Internet]. 2015 Dec [cited 2017 Nov 14];16(1). Available from: http://bmcmedethics.biomedcentral.com/articles/10.1186/s12910-015-0003-2
11. Hernández-Marrero P, Pereira SM, Carvalho AS. Ethical Decisions in Palliative Care: Interprofessional Relations as a Burnout Protective Factor? Results From a Mixed-Methods Multicenter Study in Portugal. Am J Hosp Palliat Med. 2016 Sep;33(8):723–32.
12. Hold JL. A good death: Narratives of experiential nursing ethics. Nurs Ethics. 2017 Feb;24(1):9–19.
13. Sandman L, Molander U, Benkel I. Developing organisational ethics in palliative care: A three-level approach. Nurs Ethics. 2017 Mar;24(2):138–50.
