# Supplementary material for: Real-world ethics in palliative care: A systematic review of the ethical challenges reported by specialist palliative care practitioners in their clinical practice
Source: Palliat Med. 2020 Dec 10;35(2):315–34. doi: 10.1177/0269216320974277 (PMC7897798; doi:10.1177/0269216320974277)
Supplement: sj-docx-3-pmj-10.1177_0269216320974277 – Supplemental material for Real-world ethics in palliative care: A systematic review of the ethical challenges reported by specialist palliative care practitioners in their clinical practice [file sj-docx-3-pmj-10.1177_0269216320974277.docx]

Supplementary File 3: Ethics Topic Areas in Key Textbooks

This table details the chapter headings in the ethics sections of the textbooks referenced.

| Oxford Textbook of Palliative Medicine (5^th^ Ed)^1^ | Oxford Handbook of Palliative Medicine^2^ | Textbook of Palliative Care^3^ | Introducing Palliative Care^4^ | The Ethics of Palliative Care: European Perspectives^5^ |
| --- | --- | --- | --- | --- |
| Human rights issues | **Provision of clinically assisted hydration in the dying** | **End-of-life decisions** | **Principalism** | **Good death or good life as the goal of palliative care** |
| Confidentiality | **Prognostication** | **Palliative sedation: A medical-ethical exploration** | **Respect for autonomy** | **Autonomy and palliative care** |
| Neuro-palliative care and disorders of consciousness | **Truth telling and collusion with families** | **Nutrition and hydration in palliative care and their diverse meanings** | **Doctrine of double effect** | **Palliative sedation** |
| Truth telling and consent | **Resuscitation decisions** | **Attending to the suffering other: A case study of teleconsultation in palliative care at home** | **Appropriate treatment (futility)** | **Euthanasia and physician assisted suicide** |
| Ethics in paediatric palliative care | **Capacity and consent** | **Request for assisted suicide** | **Euthanasia** | **Research ethics** |
| Dignity and palliative end-of-life care | **Euthanasia** |  |  | **Futility** |
| Euthanasia |  |  |  |  |
| Withdrawing and withholding life-sustaining treatment |  |  |  |  |

Note: This table details the top level headings for the sections/chapters within the self-identified ethics sections of each book.

1. Cherny NI, Fallon M, Kaasa S, et al. (eds). *Oxford Textbook of Palliative Medicine*. 5th ed. Oxford : Oxford University Press, 2015.

2. Watson MS. *Oxford handbook of palliative care*. [New York] : Oxford University Press, 2019.

3. MacLeod R, Van Den Block L. *Textbook of Palliative Care*. Springer International Publishing, https://doi.org/10.1007/978-3-319-31738-0 (2019, accessed 17 May 2020).

4. Twycross R. *Introducing Palliative Care*. 4th ed. Oxford: Radcliffe Medical Press, 2003.

5. ten Have H, Clark D (eds). *The ethics of palliative care: European perspectives*. Buckingham [England]; Open University Press, 2002.
